# A Natural Fungal Gene Drive Enacts Killing via DNA Disruption

**DOI:** 10.1128/mbio.03173-22

**Published:** 2022-12-20

**Authors:** Andrew S. Urquhart, Donald M. Gardiner

**Affiliations:** a Commonwealth Scientific and Industrial Research Organisation, St Lucia, Queensland, Australia; b Applied Biosciences, Macquarie University, Macquarie Park, New South Wales, Australia; c The University of Queensland, St Lucia, Queensland, Australia; Universidad de Córdoba

**Keywords:** spore killer, mechanism, fungi, DNA, gene drives, filamentous fungi, nucleases

## Abstract

Fungal spore killers are a class of selfish genetic elements that positively bias their own inheritance by killing non-inheriting gametes following meiosis. As killing takes place specifically within the developing fungal ascus, a tissue which is experimentally difficult to isolate, our understanding of the mechanisms underlying spore killers are limited. In particular, how these loci kill other spores within the fungal ascus is largely unknown. Here, we overcome these experimental barriers by developing model systems in 2 evolutionary distant organisms, Escherichia coli (bacterium) and Saccharomyces cerevisiae (yeast), similar to previous approaches taken to examine the *wtf* spore killers. Using these systems, we show that the Podospora anserina spore killer protein SPOK1 enacts killing through targeting DNA.

## INTRODUCTION

Gene drives are genetic elements that positively distort the frequency in which they are inherited. One group of meiotic gene drives are the evolutionarily diverse spore killer proteins found in ascomycete fungi. These gene drives distort normal mendelian inheritance by killing sibling progeny that do not inherit the locus (1). Spore killers include *het-s* and *Spok* in Podospora anserina (2, 3); *Sk-1*, *Sk-2* and *Sk-3* in Neurospora (4, 5), *Sk^K^* in Fusarium verticillioides (6), and *wtf* genes in Schizosaccharomyces pombe (7, 8).

Understanding how these gene drives function is important. Firstly, they likely influence genome evolution and structure, including mobile elements (9). Secondly, uncovering their mechanisms might provide insight into the conserved biological processes that they disrupt to cause killing (1). Thirdly, they are potentially useful in pathogen control strategies. Gene drive approaches have already been widely investigated as control strategies for insect pests, notably malaria-transmitting mosquitoes (10). The *Spok1* spore killer from Podospora anserina has been shown to function heterologously in the dung-colonizing fungus Sordaria macrospora (3) and the plant pathogen Fusarium graminearum (11). The genetic control of plant-pathogenic fungi would decrease the need for the environmentally damaging fungicides currently used in agriculture.

Gene drive elements show considerable diversity. For example, the Neurospora *Sk-2* and *Sk-3* drives rely on 2 separate genes, encoding separate proteins to enact killing and resistance functions (12). On the other hand, in some drive systems such as the *wtf* genes in S. pombe the killing and resistance functions are encoded by 2 separate proteins translated from alternative transcripts of the same gene (7, 8). In the case of the SPOK proteins in P. anserina, only one transcript has been detected and it is not known how this single transcript can lead to both killing and resistance (13).

For the most part, the cellular processes or structures targeted by the killing activity of spore killers is unknown. One exception is the *Het-s* locus (reviewed by [1]). Two alleles have been identified at this locus, *het-S* and *het-s*, encoding the proteins HET-S and HET-s, respectively. Killing is enacted via interaction between these 2 proteins, which induces a conformational change in the HET-S protein exposing a previously buried transmembrane domain. The altered HET-S protein perforates cell membranes, killing the cell (14). Another is the *wtf4* gene drive which forms toxic protein aggregates in Schizosaccharomyces pombe (15).

As with most other spore killers, the mechanism by which SPOK proteins kill the developing gametes which did not inherit it is currently unknown. Bioinformatic analysis suggests that SPOK proteins consists of 3 domains (13). Experimental evidence suggests that the second domain, a putative nuclease, is required for killing activity and the third domain, a putative kinase is required for resistance. Specifically, a mutation (D667) within the third domain of SPOK3 results in an allele which could not be transformed into P. anserina, suggesting that it was toxic even in vegetative tissue (13). On the other hand, another mutation (K240) within the predicted catalytic core of the putative nuclease domain was found to abolish killing activity but not resistance (13). Vogan et al. conjectured that a possible mode of action for the nuclease domain was the synthesis of a toxic diffusible metabolite (13).

A key difficulty in uncovering the mechanisms underlying spore killing is the limited availability of the relevant tissue (developing fungal asci). Indeed, in the case of *het-S*, the mechanism was largely uncovered through examining its role in heterokaryon incompatibility, negating the need to examine developing asci. On the other hand, the mechanisms of the *wtf4* gene drive were uncovered using a heterologous system in S. cerevisiae (15). Similarly, to overcome this issue, we sought to examine the activity of *Spok1* in the experimentally amenable organism Escherichia coli. We here present evidence that the killing activity of SPOK1^D680A^ protein (equivalent to SPOK3^D667A^) is indeed active in E. coli, furthermore that this activity is mediated via the disruption of the chromosomal DNA and use the eukaryotic system of S. cerevisiae to infer a mechanism involving DNA damage. Given that the killing activity is known to depend on a domain with similarity to restriction endonucleases, we suggest that direct modification (for example cleavage) of the genomic DNA by the nuclease domain represents a likely mechanism.

## RESULTS

### A *Spok1* allele carrying a mutated resistance domain (*Spok1*^D680A^) is toxic to E. coli.

**FIG 1:**
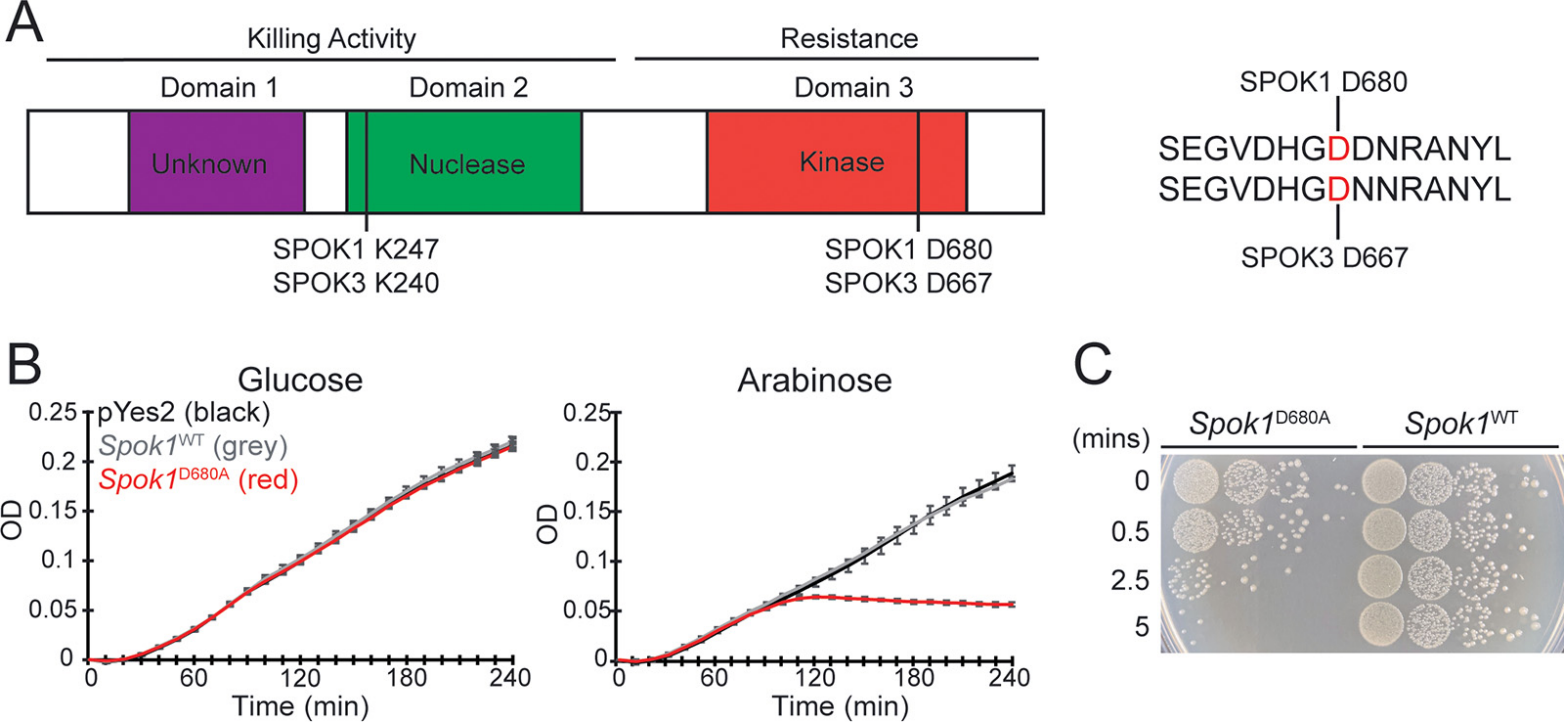
(A) Representation of the domain structure of SPOK proteins, according to (13). The spore killing activity of the SPOK proteins requires the nuclease (second) and possibly first domains. Host resistance is mediated by the third domain, a putative kinase. Experimental evidence suggests that mutation of aspartic acid (D) 667 to alanine (A) in SPOK3 results is a toxic allele possessing only killing activity (13). Protein alignments showed that this corresponds to residue D680 in SPOK1. (B) Growth curve of E. coli expressing *Spok1*^WT^ and *Spok1*^D680A^ after induction by arabinose. Error bars represent ± 1 standard deviation. pYes2 is an empty vector expressing no SPOK1 protein. (C) 10-fold dilution series of induced cells at various time points after induction passaged back onto high glucose concentrations and incubated for 24 h to allow colonies to develop.

Inspired by the toxic version of SPOK3 being functional in vegetative cells of P. anserina (12), we reasoned that killing may target a conserved biological process and sought to identify a highly tractable system in which toxicity could be assessed. Such a system requires tightly regulable gene expression, which in E. coli can be achieved with the arabinose-inducible and glucose-repressible P_BAD_ promoter (16). A protein alignment revealed that the SPOK3 residue D667 previously found to be essentially for resistance activity (13) corresponds to residue D680 in SPOK1 (Fig. 1A) and we created the equivalent mutated allele in *Spok1* (hereafter termed *Spok1*^D680A^). When cloned under the control of the P_BAD_ promoter, the wildtype and toxic version of *Spok1* grew similarly under repressive (high glucose) conditions. Expression of *Spok1*^D680A^ caused E. coli growth to stop approximately 100 min after arabinose induction (Fig. 1B). In contrast, cells expressing *Spok1*^WT^ continue to grow as do those containing an empty vector control (pYes2). A double mutant allele in which an additional residue, K247 corresponding to K240 of SPOK3, within the nuclease domain previously shown to be essential for killing was mutated had minimal effect on growth rate as did a single mutant in K247 (Fig. S1). This effect was observed in 2 commonly used E. coli strains Top10F’ (arabinose non utilizing) and DH5α (arabinose utilizing).

10.1128/mbio.03173-22.1 FIG S1 (A) Growth curve of E. coli expressing four different Spok1 alleles Spok1^WT^, Spok1^K247A^ Spok1^D680A^ or Spok1^K247A/D680A^ after induction by arabinose. (B) OriC:ter ratio 2.5 h after arabinose induction shows that only Spok1^D680A^ expression elevates the oriC:ter ratio. (C) PFGE analysis of E. coli expressing the four Spok1 alleles shows that only Spok1^D680A^ expression leads to increased chromosomal fragmentation. Download FIG S1, TIF file, 2.2 MB. Copyright © 2022 Urquhart and Gardiner. 2022 Urquhart and Gardiner. https://creativecommons.org/licenses/by/4.0/ This content is distributed under the terms of the Creative Commons Attribution 4.0 International license.

We next sought to determine if the expression of *Spok1*^D680A^ was merely an inhibitor of growth or a genuine killer. To this end we attempted to rescue arabinose induced cells by rapidly diluting them into high concentrations of glucose to suppress gene expression. This assay showed that the E. coli cells expressing *Spok1*^D680A^ quickly lose viability after induction with an almost complete loss of viability after just 5 min in inducive conditions (Fig. 1C).

### Genes proximal to the origin of replication show elevated expression in response to killing.

**FIG 2:**
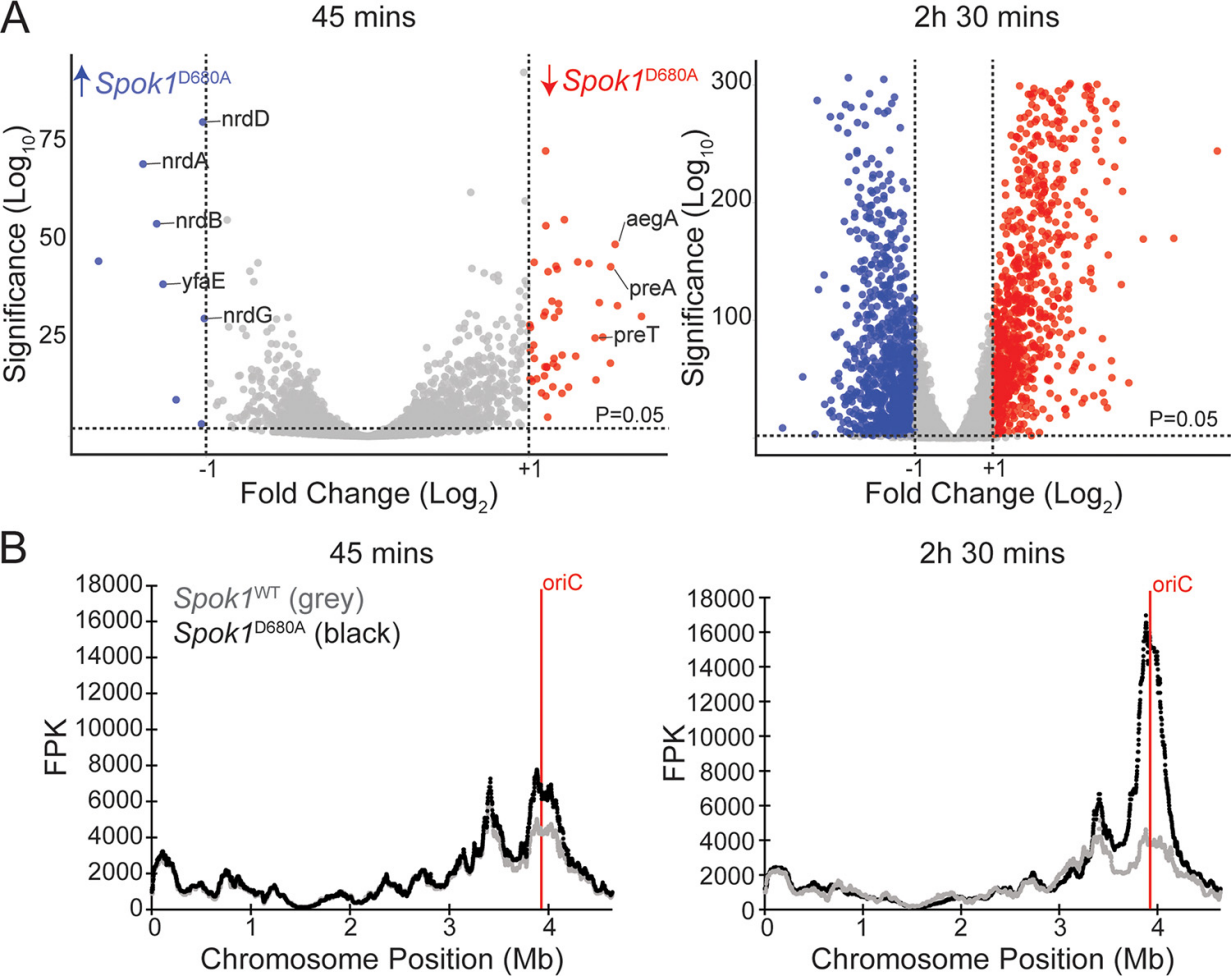
(A) Volcano plots showing RNA seq data at 45 min and 2 h 30 min. Blue genes are more highly expressed in E. coli expressing *Spok1*^D680A^ and red genes are more highly expressed (greater than 2-fold change) in E. coli expressing *Spok1*^WT^. Specific genes involved in DNA metabolism that are differentially regulated at 45 min post induction are annotated. (B) Median gene expression (100 gene windows) along the E. coli chromosome. Increased expression in genes proximal to the *oriC* in E. coli expressing *Spok1*^D680A^ (compared to cells expressing *Spok1*^WT^) is observed at both 45 min and 2 h 30 min but is more pronounced at the latter time point.

To determine the mechanisms underlying the killing in E. coli, we conducted RNA sequencing on induced E. coli expressing either *Spok1*^WT^ or *Spok1*^D680A^. Time points for RNA sequencing were chosen based on the OD curves rather than the rescue assay as OD readings are likely to better reflect the timing of physiological changes in the cell, whereas the rescue assay measures the time point at which the *Spok1*^D680A^-expressing cells were committed to death, but not yet dying. At an early time point (45 min), expression changes were relatively minor compared to the later time point (2 h 30 min) (Fig. 2A). Only 55 genes showed statistically significant (at *P* < 0.05) and greater than 2-fold change at 45 min (Table S2) compared to 1076 genes at 2 h 30 min.

10.1128/mbio.03173-22.3 TABLE S2 DEseq2 output for differentially regulated genes. Download Table S2, XLSX file, 0.01 MB. Copyright © 2022 Urquhart and Gardiner. 2022 Urquhart and Gardiner. https://creativecommons.org/licenses/by/4.0/ This content is distributed under the terms of the Creative Commons Attribution 4.0 International license.

Examination of the 55 genes showing altered regulation at 45 min revealed at least 8 genes involved in nucleotide metabolism including *nrdA*, *nrdB*, *nrdD*, *nrdG*, *yfaE*, *aegA*, *preA*, and *preT*. Four of these genes (which were all upregulated in the *Spok1*^D680A^ expressing strain) encode subunits of the 2 E. coli ribonucleotide reductases (RNR). RNRs convert ribonucleotides to deoxyribonucleotides which are essential for DNA synthesis. NrdA and NrdB form the aerobic RNR in E. coli and the neighboring gene *yfaE* may play a role in the functioning of this enzyme (17). NrdD and NrdG form the anaerobic RNR (18). The downregulated genes included genes required for the degradation of pyrimidines and purines. Namely, PreA and PreT catalyze the reduction of uracil to 5,6-dihydrouracil in the breakdown of pyrimidine bases (19). AegA is involved in the breakdown of purine nucleotides through the degradation of urate (20).

By the 2 h 30 min time point the extent of gene expression changes rendered the identification of individual genes impractical (Fig. 2A). However, mapping gene expression along the chromosome revealed an upregulation in genes surrounding the origin of replication in the strain expressing *Spok1*^D680A^ relative to the strain expressing *Spok1*^WT^ (Fig. 2B). This difference was already present at 45 min but more pronounced at 2 h 30 min.

### *Spok1*^D680A^ expression interferes with DNA metabolism.

**FIG 3:**
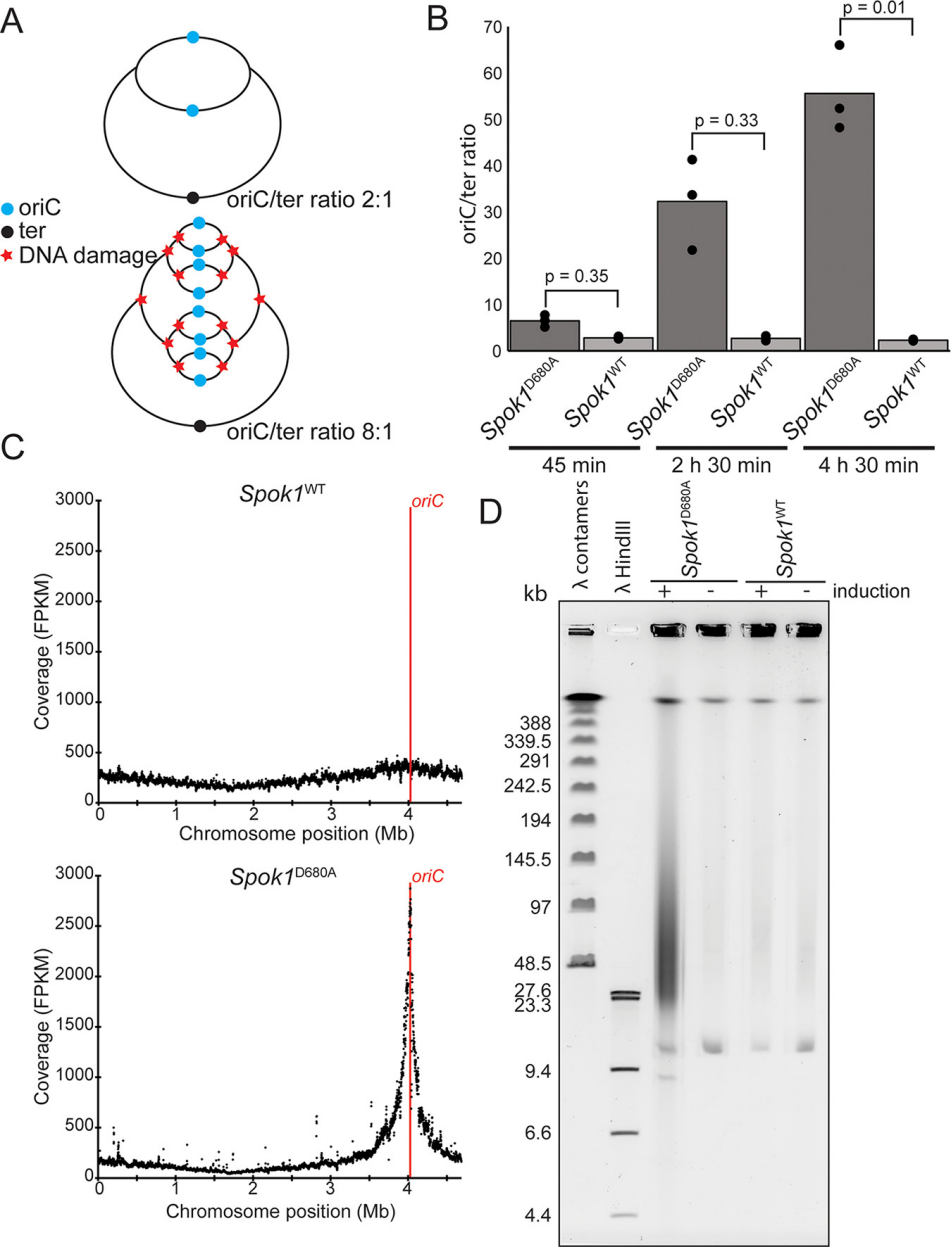
(A) Diagram displaying the effect of DNA damage on *oriC*/*ter* ratio. If DNA damage prevents the replication form progressing fewer replication forks will reach the terminus of the chromosome. As replication forks continue to be initiated but fail to complete replication the ratio between *oriC* and *ter* DNA will increase. (B) qPCR analysis of the *oriC*/*ter* ratio following induction of either *Spok1*^WT^ or *Spok1*^D680A^. DNA sequencing read depth compared to chromosomal position at 2 h 30 min following induction of either *Spok1*^WT^ or *Spok1*^D680A^. (C) Read depth (FPKM) of Illumina DNA sequencing reads mapped to the E. coli chromosome, each point represents a 1 kb genomic window. (D) PFGE analysis of chromosomal fragmentation at 2 h 30 min post induction. Degraded DNA appears as a smear down the gel with intact circular DNA remaining in the well.

DNA replication in E. coli proceeds bidirectionally from the origin of replication (*oriC*) to the terminus (*ter*) and new replication forks are initiated before previous rounds of DNA replication have completed, meaning that in actively growing cells there will be more copies of the DNA closer to the initiation of the DNA replication fork (Fig. 3A) (21). We hypothesized that apparent upregulation of gene expression surrounding the origin of replication was a result of a corresponding increased DNA copy number in a gradient from the *oriC* to *ter* loci. We therefore determined the ratio of cellular DNA between the *oriC* and *ter* loci, in actively growing cells, using quantitative PCR (qPCR) (Fig. 3B). This revealed that induction of *Spok1*^D680A^ resulted in an *oriC*/*ter* ratio of approximately 50:1 compared to approximately 2:1 in the strain expressing *Spok1*^WT^. The increase in DNA copy number surrounding the origin was confirmed by whole-genome DNA sequencing (at 2 h 30 min) as this showed increased relative copy number in a region of approximately 1 million bases peaking at the origin of replication (Fig. 3C). At 2h 30 min damage to the E. coli chromosome could be directly observed using PFGE electrophoresis (Fig. 3D). In this assay, circular intact chromosomes remain in the well and degraded fragments (now linear) run as a smear (reviewed by [22]). The PFGE experiment was repeated three times independently with similar results. The observation of fragmented DNA is consistent with both double-stranded breaks or other damage which results in heat labile DNA (23). No effect on *oriC/ter* ratio or chromosomal fragmentation was observed in alleles containing a mutated nuclease domain confirming the importance of this domain in killing (Fig. S1)

### SPOK1 also targets DNA to enact killing activity in a eukaryote.

**FIG 4:**
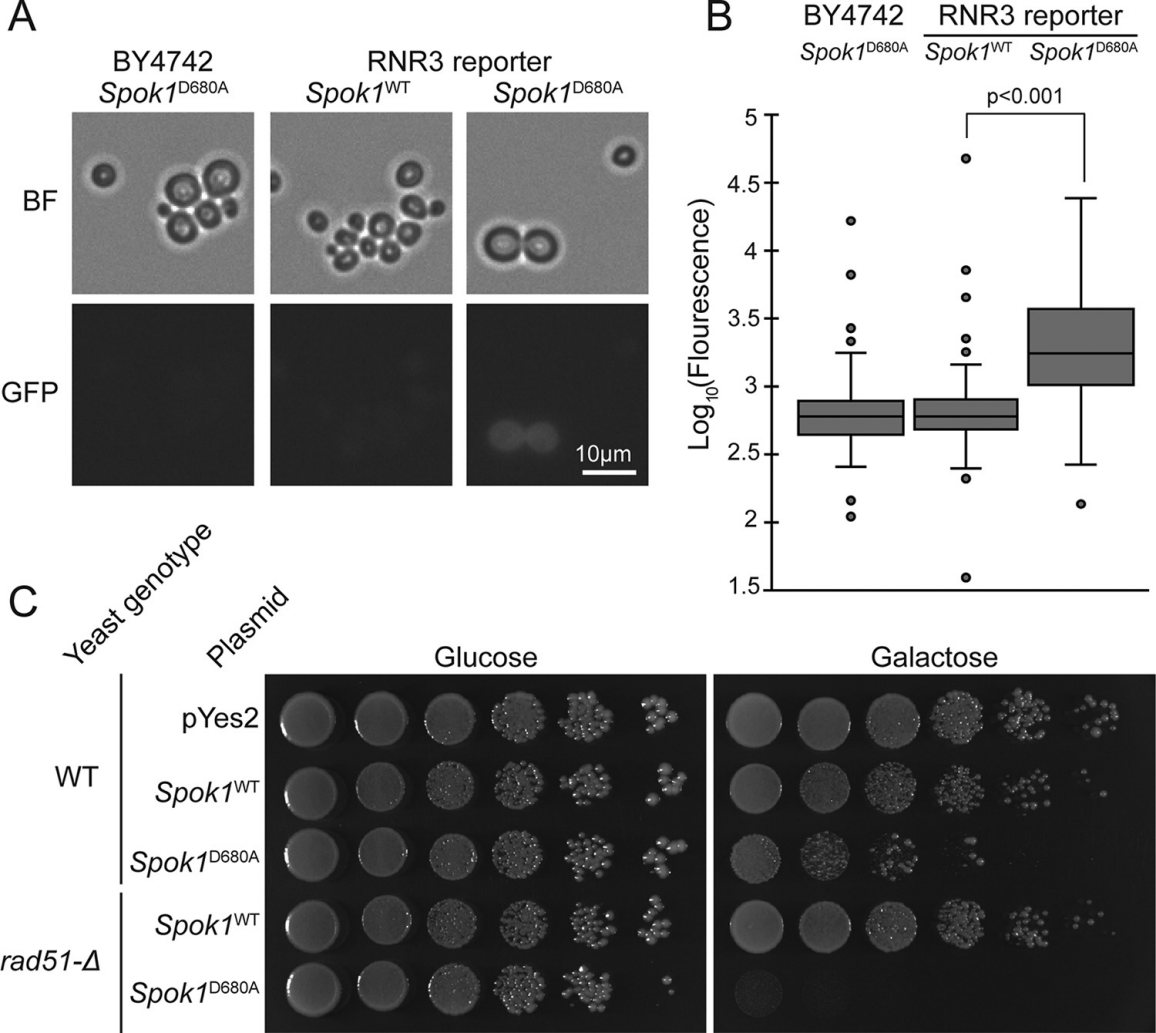
(A and B) Expression of *Spok1*^D680A^ in a DNA damage responsive RNR3:GFP reporter strain induced expression of GFP. Data points represent fluorescence intensity of induvial cells (*n* = 90). (C) Spot assay of S. cerevisiae WT and *rad51*Δ expressing *Spok1*^WT^ or *Spok1*^D680A^ under a galactose inducible promoter. Cells were passage from glucose media onto either glucose or galactose media and the amount of growth following incubation at 30°C was observed after 72 h. The *rad51*Δ mutant was hypersensitive to *Spok1*^D680A^ expression. pYes2 is an empty control vector which expresses no SPOK protein.

While E. coli is a tractable system for analyzing the mechanisms of the SPOK proteins, there are fundamental differences in the cell biology between the fungi in which *Spok* genes are found and prokaryotes, not least the enveloped nucleus in eukaryotes in which the Spok proteins are thought to reside (3). To understand if our identification of DNA as the target of SPOK proteins also occurred in eukaryotes we utilized baker’s yeast as it lacks endogenous copies of Spok genes but shares many fundamental aspects of eukaryote biology with filamentous ascomycetes. Indeed, expression of *Spok1*^D680A^ in S. cerevisiae reduced growth after 3 days incubation (Fig. 4C). Although much reduced in response to *Spok1*^D680A^ expression, growth continued over the next 14 days suggesting that the effect does not kill the cells outright (data not shown).

To further support that DNA was a target of *Spok1*, we sought to assay whether *Spok1*^D680A^ was triggering DNA damage in yeast. To do this, we developed an RNR3:GFP strain to monitor expression of *RNR3* which is known to be responsive to DNA damage such as hydroxyurea as previously reported (data not shown) (24). Induction of *Spok1*^D680A^ in this reporter strain (Fig. 4A and B) demonstrates upregulation of *RNR3* compared to expression of *Spok1*^WT^ which presumably is in response to DNA damage. Increased autofluorescence was not observed in a WT control strain upon expression of *Spok1*^D680A^.

Having established that DNA damage was most likely also occurring when *Spok1*^D680A^ was expressed, we hypothesized that mutants impaired in their ability to repair DNA damage would show hypersensitivity to *Spok1*^D680A^ expression. Rad51 deletion mutants show increased sensitivity to DNA damaging agents with Rad51 acting as a DNA recombinase in pathways for repairing DNA damage (25). As expected, the *rad51*Δ mutant to showed hypersensitivity to *Spok1*^D680A^ expression (Fig. 4C).

## DISCUSSION

The mechanisms underlying the gene drive mechanisms of fungal spore killers are poorly understood. We here provide several lines of evidence that the SPOK1 protein of *Podospora* acts through DNA damage. Given the complexity of the relevant tissue (developing fungal ascus in the context of structural components of the perithecia) we employed heterologous model systems. We demonstrated that a mutated version of the SPOK1 protein lacking resistance function (SPOK1^D680A^) was toxic in the model organisms E. coli and S. cerevisiae (Fig. 1B and 4A). These systems provide obvious experimental advantages over examining meiotic drive *in situ* during fungal crosses. Because the protein was functional in organisms as evolutionary distant as E. coli and yeast, we hypothesized that *Spok* killing activity must target a highly conserved structure/pathway. Furthermore, given the conservation of key residues between SPOK1 and other SPOK proteins, presumably the mode of action described here will be conserved in this gene family.

Firstly, we took a mechanism-neutral approach in E. coli and conducted RNA sequencing on E. coli cultures induced to express *Spok1*^D680A^. At an early time point (45 min), several genes involved in DNA metabolism were differentially regulated (Fig. 2A). These included several RNR subunits. E. coli RNR genes are known to be regulated in response to DNA damage (26). Of note is that the RNA sequencing analysis was conducted in a *recA* mutant (recA is a homolog of yeast RAD51) for improved plasmid stability. RecA is an important regulator of DNA repair pathways so many DNA damage inducible genes will be unresponsive in this strain (27). The altered regulation of genes involved in DNA metabolism at 45 min post induction provided initial evidence that DNA metabolism was disrupted during SPOK1 killing.

Examination of a later time point (2 h 30 min post induction) showed an unexpectedly strong expression of genes within a region of ~1Mbp centered around the origin of replication. Gene expression levels are known to correlate with chromosomal position in E. coli with highest expression closest to the origin (28). However, in E. coli expressing *Spok1*^D680A^ this effect was amplified (Fig. 2B).

With evidence for specific DNA metabolism related genes being regulated at the 45 min time point, we hypothesized that this effect might be due to an underlying perturbation in corresponding DNA copy number. The circular E. coli chromosome replicates bidirectionally from the origin to the terminator. Because new replication forks are initiated before previous replication forks have completed this results in a ratio of *oriC*/*ter* greater than one in actively dividing cells (29). Treatments which block the progression of the replicon e.g., hydroxyurea (which inhibits RNR and damages DNA) or antibiotics such as trimethoprim (which inhibit DNA synthesis) are known to increase the *oriC*/*ter* ratio (30, 31). qPCR and whole-genome Illumina sequencing confirmed that the RNA-seq expression patterns were a result of DNA perturbations (Fig. 3). Further supporting the DNA perturbations as the cause of killing PFGE analyses demonstrated increased DNA fragmentation upon induction of *Spok1*^D680A^ expression. While fragmentation of chromosomal DNA might be an expected eventual consequence of cell death the fact that we see elevation in the *oriC/ter* ratio in the mRNA as well as the DNA demonstrates that this distortion occurs while the cell is still metabolically active (i.e., still transcribing mRNA). Additionally, post death chromosomal degradation would likely be random across the chromosome and not influence *oriC/ter* ratio. Further support for a primary role of DNA disruption comes from the altered regulation of genes related to DNA metabolism at an early point, as well as the fact that killing is dependent of the nuclease domain (Fig. S1).

Given the evolutionary distance between bacteria and the eukaryotes in which SPOK proteins occur natively, we decided to induce expression of *Spok1*^D680A^ in the yeast S. cerevisiae. As in E. coli, expression of SPOK1 protein proved toxic to the yeast cells. We took 2 difference approaches to demonstrate that the killing of S. cerevisiae occurred through genotoxic stress. The first was to express *Spok1*^D680A^ in an S. cerevisiae RNR3:GFP reporter strain. Rnr3 is a ribonucleotide reductase gene that is expressed in response to DNA damage (24). Induction of *Spok1*^D680A^ induced GFP fluorescence in this strain (Fig. 4A and B). Secondly, we expressed *Spok1*^D680A^ in a *rad51*Δ mutant. This mutant is known to be deficient in DNA repair and was hypersensitive to *Spok1*^D680A^ (Fig. 4C). Increased sensitivity in a DNA repair deficient yeast strain is further evidence for a primary role of DNA disruption (as opposed to post death chromosomal degradation).

A limitation of this study is that we have not examined the activity of SPOK1 protein *in situ* during fungal crosses to demonstrate the occurrence of DNA damage within a fungal ascus, as such an experiment is technically challenging. That the D680A mutation behaves in the same way in both E. coli and S. cerevisiae, with evidence of DNA stress occurring in both species, suggests that the same mode of action will occur in the fungal ascus. The common response to *Spok1*^D680A^ expression in these 2 distantly related species is consistent with a highly conserved molecular target, such as genomic DNA.

The exact mechanism underlying the effect of SPOK1^D680A^ on DNA is still unclear. The killing activity of SPOK1 proteins has previously been shown to require a catalytically active nuclease domain with similarity to type I restriction endonucleases (13). Also consistent with DNA targeting, Grognet et al. showed that Spok proteins localize to the fungal nucleus in developing asci (3). We thus suggest that the simplest mechanism for *Spok1* killing, consistent with our data, nuclear localization and the domain content of the protein, would be cleavage (or other direct damage) of the chromosomal DNA by the nuclease domain of SPOK1. Other toxins are known to work via enzymatic DNA damage for example Cytolethal distending toxin (CDT) produced by certain bacterial pathogens causes double-stranded DNA breaks in mammalian cells (32).

There are parallels to bacterial restriction modification (RM) systems which are known to behave as selfish elements (33). They consist of a restriction enzyme and a methyltransferase that methylates the target site and, thus, protects the genome. However, if the selfish RM element is lost from the cell (e.g., if carried on an unstable plasmid) then the cell is killed (reviewed by [34]). This is curiously reminiscent of fungal gene drives in which spores not inheriting the drive element are killed. However, in the case of SPOK proteins, the resistance activity is encoded by a putative kinase domain of the same protein, not a separate methyltransferase enzyme, so the mechanism underlying resistance remains unclear. We envisage that the E. coli and S. cerevisiae models reported here will enable further studies into the activity of Spok proteins including how resistance is mediated.

## MATERIALS AND METHODS

### Strains used.

The strains used in this study include: (i) Escherichia coli DH5α: F^–^ *endA1 glnV44 thi-1 recA1 relA1 gyrA96 deoR nupG purB20* φ80d*lacZ*ΔM15 Δ(*lacZYA-argF*)U169, hsdR17(*r_K_*^–^*m_K_*^+^), λ^–^. (ii) E. coli Top10F’: F'[*lacI*^q^Tn10(Tet^R^)] *mcrA* Δ(*mrr*-*hsdRMS-mcrBC*) φ80*lacZ*ΔM15 Δ*lacX74 recA1 araD139* Δ(*ara-leu*)*7697 galU galK rpsL endA1 nupG*. (iii) Saccharomyces cerevisiae BY4742: MATα *his3*Δ1 *leu2*Δ0 *lys2*Δ0 *ura3*Δ0.

### Molecular cloning.

**(i) E. coli expression constructs.** Plasmids were generated to express *Spok* genes (PLAUB44 *Spok1*^D680A^ or PLAUB51 *Spok1*^WT^) under the arabinose-inducible P_BAD_ promoter (16). The *araC*-P_BAD_ fragment was amplified from the genomic DNA of E. coli BL21(DE3) using primers AUB283 + AUB284. Two fragments of the yeast plasmid pYES2 backbone were amplified using primers AUB287 + DG1289; and primers DG1290 + AUB288. *Spok1*^WT^ was amplified using primers AUB285 + AUB286 and *Spok1*^D680A^ was amplified in 2 fragments with AUB285 + AUB236 and AUB237 + AUB286. Oligonucleotide sequences are provided in Table S1. All PCRs were conducted using Q5 High-Fidelity 2X PCR Master Mix following the manufacturer’s directions (New England Biolabs). In order to assemble these fragments together, they were transformed into S. cerevisiae by heat shock using a standard lithium acetate method (35) and were combined into a single plasmid via homologous recombination in the yeast cells (36). The plasmid was rescued into chemically competent E. coli using the Zymoprep Yeast Plasmid Miniprep Kit (Zymo Research). Competent cells were prepared using the Inoue method (37).

10.1128/mbio.03173-22.2 TABLE S1 Oligonucleotides designed in this study. Download Table S1, DOCX file, 0.01 MB. Copyright © 2022 Urquhart and Gardiner. 2022 Urquhart and Gardiner. https://creativecommons.org/licenses/by/4.0/ This content is distributed under the terms of the Creative Commons Attribution 4.0 International license.

**(ii) S. cerevisiae expression construct.** *Spok1* coding DNA was amplified from PLAUB44 (*Spok1*^D680A^) or PLAUB51 (*Spok1*^WT^) (11) using AUB516 + AUB517 and cloned into the HindIII site of pYES2 using the NEBuilder HiFi DNA Assembly Master Mix (New England Biolabs) following the manufacturer’s directions. The primers were designed to include an optimal translation initiation site, including a synonymous C to T mutation in the second codon. This construct results in either *Spok1*^WT^ or *Spok1*^D680A^ expression under the galactose inducible *GAL1* promoter of pYES2.

### E. coli assays.

E. coli containing the *Spok1* plasmids were maintained on LB + carbenicillin + 0.2% glucose. For induction of *Spok1* expression, the E. coli was first grown overnight in 5 mL LB + carbenicillin + glucose at 37°C with shaking. A total of 50 μL of overnight culture was used to inoculate 50 mL of LB + carbenicillin in a 250 mL Erlenmeyer flask and allowed to grow for a further 4 h. At this point, arabinose or glucose was added to 0.2% and incubation continued at 37°C either in the flask (for nucleic acid extraction or PFGE) or diluted 1:1 with fresh LB media and incubated in a 96-well plate to acid monitor growth in a plate reader. Due to the faster growth rate of NEB Turbo cells this strain was diluted 1:10 rather than 1:1 with fresh LB. The plate reader assay was conducted in an EnVision Multimode Plate Reader with shaking (5 s, 900 rpm, 0.1 mm diameter, linear) every 10 min before reading OD at 595 nm.

To determine if *Spok1*^D680A^ expression merely arrested growth or effected permanent killing, we attempted to “rescue” the strains at various time points after arabinose induction. This was done by pipetting 10 μL of induced culture into 1 mL of LB + glucose then plating out onto LB + glucose + carbenicillin agar plates.

### Pulse field gel electrophoresis.

E. coli were induced for 2.5 h as described above and then harvested and resuspended to OD 10 in resuspension buffer (100 mM Tris, 1 mM EDTA, pH 8). A total of 20 μL of 20 mg/mL proteinase K was added to 400 μL of cellular suspension and then mixed with an equal volume of 1% SeaKem Gold agarose in TE (50°C) and set in plug molds as described previously (38). Plugs were lysed in lysis buffer (50 mM Tris, 50 mM EDTA, 1% Sarcosyl, pH 8) for 2 h and 50°C, then washed twice (10 min each) with TE before loading into a 1% SeaKem Gold agarose/0.5X TBE gel. Pulse field electrophoresis was carried out using a Bio-Rad CHEF-DR III system (12°C, 6 V/cm, switching time 2 to 28.6 s, 120^°^ angle, 14 h run time, 0.5X TBE buffer).

### RNA sequencing.

E. coli strains containing plasmids PLAUB44 (*Spok1*^D680A^) or PLAUB51 (*Spok1*^WT^) were induced with arabinose as described above. RNA was extracted from 5 mL of E. coli culture at 45 min and 2 h 30 min, after induction for RNA sequencing. Four replicates for each construct at each time point were analyzed (16 samples total). E. coli cultures were pelleted by centrifugation and then lyophilized before DNA was extracted using TRIzol reagent following the manufacturer’s instructions. The RNA was sequenced at the Australian Genome Research Facility. The libraries were prepared using the Illumina Stranded Total RNA Prep Ligation with Ribo-Zero Plus kit and sequenced on an Illumina NovaSeq 6000.

RNA sequence analysis was conducted using Galaxy (39). Reads were mapped to the E. coli chromosome using STAR (40), reads mapping to each gene were counted using featureCount (41) and differentially expressed genes were determined using DEseq2 (42).

### DNA sequencing data and qPCR.

A total of 20 mL of E. coli cultures were pelleted by centrifugation and then lyophilized before DNA was extracted using the Qiagen Plant minikit. DNA was further purified by an ethanol precipitation step followed by resuspension in pure water. DNA quality was confirmed by gel electrophoresis and a nanodrop spectrophotometer. DNA was extracted at 45 min, 2 h 30 min, 4 h 30 min (3 biological replicates each) post arabinose induction for E. coli transformed with PLAUB44 (*Spok1*^D680A^) or PLAUB51 (*Spok1*^WT^).

The *oriC*/*ter* ratio was determined using a hydrolysis probe based duplex qPCR assay designed to conform with the MIQE guidelines (43). Primers TTCGATCACCCCTGCGTACA and CGCAACAGCATGGCGATAAC amplified part of the *gidA* gene located close to the origin (44). This product was detected using a FAM labeled probe AUB458 6-FAM/ATGAGTGAT/ZEN/ATAACACGGCACCTGCTGG/IBFQ (IDT). Primers AUB484 + AUB485 were used to amplify part of the *dcp* gene located near the terminus. This product was detected using a Cy5 labeled probe Cy5/AACCCGCCC/TAO/TGCTGCTTATCGATAAC/IBRQ (IDT). qPCRs were conducted using Luna Universal Probe qPCR mastermix (New England Biolabs). Reactions were set up in a total volume of 10 mL containing 0.4 μM each probe, 0.2 μM each primer and approximately 100 ng of template DNA. Reactions were run on a Bio-Rad CFX384 qPCR machine (initial denaturation of 95°C for 10 min followed by 95°C 10 sec, 58°C 10 sec, 72°C 15 sec for 40 cycles). The PCR efficacy was calculated using a 10-fold dilution series of DNA extracted from WT cells in late stationary phase (72 h). The *oriC*/*ter* of stationary phase cells is expected to be near 1 (29, 45, 46). *gidA* was found to amplify at 98.3% efficiency and *dcp* at 97.4% efficiency. Given the similar PCR efficiency between the 2 targets we did not adjust calculations to account for reaction efficiency.

The effect of *Spok1*^D680A^ expression on the E. coli chromosome was further explored via short-read whole-genome sequencing. DNA samples extracted from E. coli expressing *Spok1*^WT^ and *Spok1*^D680A^ 2 h 30 min after induction were sequenced on a NovaSeq 6000 generating 150 bp paired end reads at the Victorian Clinical Genetics Services. The resultant reads were mapped to the E. coli chromosome using Bowtie 2 (47) in Galaxy, coverage across the chromosome was calculated from the resultant BAM file using bamCoverage (48) using 1 kb windows.

### *RAD51* deletion in S. cerevisiae.

The *LEU2* selectable marker was amplified from plasmid pGAD-c1 (49) using primers AUB528 + AUB529. The resultant PCR product was used to delete the *RAD51* gene via homologous recombination in S. cerevisiae strain BY4742. Transformation was conducted using LiAc/PEG (35) and deletion of the gene was confirmed using primers AUB488 + AUB489, which amplify across the mutated region.

### S. cerevisiae growth assay.

To determine the effect of *Spok1*^D680A^ expression in S. cerevisiae a spot assay on agar plates was employed. Strains carrying PLAUB90 (*Spok1*^WT^) or PLAUB91 (*Spok1*^D680A^) were streaked on SD without uracil with 2% glucose or galactose and grown for 48 h at 30°C. Colonies were picked into sterile water, serially diluted, and plated out onto SD without uracil with 2% glucose or galactose plates.

### *RNR3* promoter GFP reporter strain.

GFP was introduced into the S. cerevisiae *RNR3* locus. GFP was amplified from plasmid PLAU17 (50) with primers AUB536 + AUB537 the *LEU2* gene was amplified with primers AUB538 + AUB539 from plasmid pGAD-c1 (49). Both products were simultaneously transformed into S. cerevisiae strain BY4742. Transformants were screened for hydroxyurea (HU) inducible GFP expression (SC with 50 mM HU versus no HU). GFP expression was quantified using a Biotek Cytation 1 plate imager at 12 h hours post induction on a microscope slide. GFP signals were captured using the Biotek GFP filter cube (469/525) with excitation using the GFP LED with an emission peak at 465 nm. The fluorescence of individual cells was quantified using Image J software. Cells were pelleted via centrifugation and resuspended in water before examination to minimize background fluorescence.

The *Spok1* plasmids were introduced into the RNR3-GFP strain and GFP fluorescence was measured following 12 h growth in SD without uracil with galactose.

### Data availability.

RNA and DNA sequencing reads are available under BioProject PRJNA798172.
