# Supplementary figures and images for: A Natural Fungal Gene Drive Enacts Killing via DNA Disruption
Source: mBio. 2022 Dec 20;14(1):e03173-22. doi: 10.1128/mbio.03173-22 (PMC9972908; doi:10.1128/mbio.03173-22)

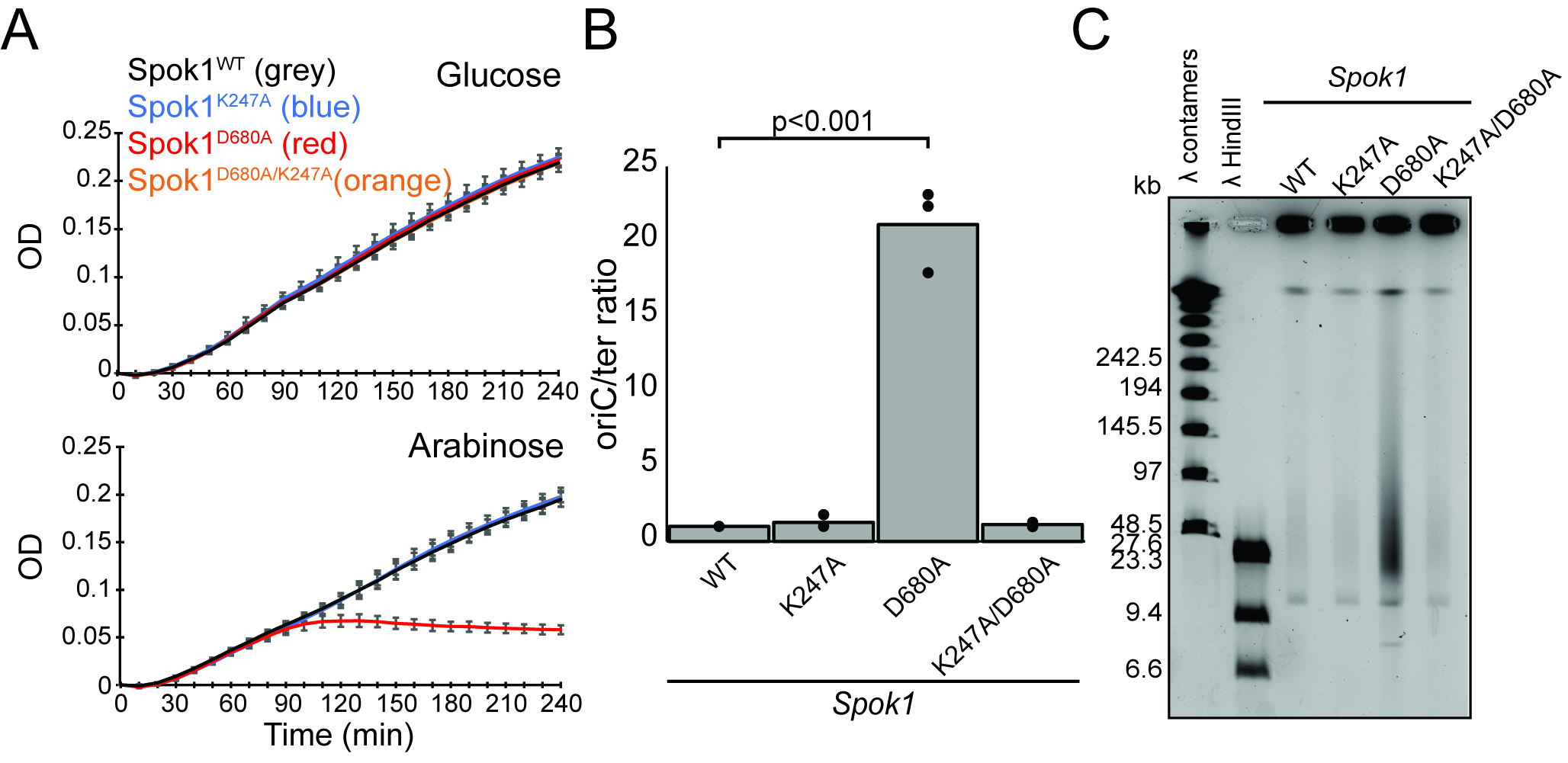

Supplement: FIG S1 [file mbio.03173-22-s0002.tif]
