# Supplementary material for: A Natural Fungal Gene Drive Enacts Killing via DNA Disruption
Source: mBio. 2022 Dec 20;14(1):e03173-22. doi: 10.1128/mbio.03173-22 (PMC9972908; doi:10.1128/mbio.03173-22)
Supplement: TABLE S1 [file mbio.03173-22-s0003.docx]

| Oligonucleotide | Sequence (5′ to 3′) |
| --- | --- |
| AUB283 | GCTTAATGGGGCGCTACAGTGCATAATGTGCCTGTCAAATGG |
| AUB284 | GGTATATCTCCTTCTTAAAGTTAAACAAAATTATTTCTAGAGCCAAAAAAACGGGTATGGAG |
| AUB285 | CTCTAGAAATAATTTTGTTTAACTTTAAGAAGGAGATATACCATGTCCGACAAAGACAGGATTG |
| AUB286 | CGATTCATTAATGCAGGGCCGCCTGTCGTAACACCTTATAC |
| AUB287 | GTATAAGGTGTTACGACAGGCGGCCCTGCATTAATGAATCG |
| AUB288 | CCATTTGACAGGCACATTATGCACTGTAGCGCCCCATTAAGC |
| AUB236 | GATAATTGGCCCTATTGTCCGCCCCATGATCAACACCCTCGC |
| AUB237 | GCGAGGGTGTTGATCATGGGGCGGACAATAGGGCCAATTATC |
| DG1289 | AAAGGACAAGGACCTGAGCG |
| DG1290 | ACTATACTAGATACTCCGTCTACTGT |
| AUB516 | ACTCACTATAGGGAATATTAACACAATGTCTGACAAAGACAGGATTGC |
| AUB517 | TGGATCCGAGCTCGGTACCATCAAGTACGAATTTGGTGTCTCG |
| AUB484 | TTCGCGGTCCAGAATACACC |
| AUB485 | CGAGCGATGAATTAGCCTCC |
| AUB528 | GTAGTTATTTGTTAAAGGCCTACTAATTTGTTATCGTCATAACTGTGGGAATACTCAGG |
| AUB529 | TAAACCTGTGTAAATAAATAGAGACAAGAGACCAAATACTTAAGCAAGGATTTTCTTAAC |
| AUB488 | TACTAGTAGTTGAGTGTAGC |
| AUB489 | AAAATGTACGGAACGCAACC |
| AUB536 | AAGAATAGCAGCAGCAATAAATCAAATACTCCCACACAAATGGTGAGCAAGGGCGAGGAG |
| AUB537 | CATAAATCATAAGAAATTCGCTCACTTGTACAGCTCGTCCATG |
| AUB538 | CATGGACGAGCTGTACAAGTGAGCGAATTTCTTATGATTTATG |
| AUB539 | GTTAGATAAGGAAAGGGAAAAATGCCACCAGAAAGAAAACTGTGGGAATACTCAGGTATC |
